# Supplementary material for: Simultaneous Stimulation of Peripheral Blood Mononuclear Cells with CpG ODN2006 and α-IgM Antibodies Leads to Strong Immune Responses in Monocytes Independent of B Cell Activation
Source: Cells. 2024 Nov 5;13(22):1822. doi: 10.3390/cells13221822 (PMC11593001; doi:10.3390/cells13221822)
Supplement: Supplementary file 1 [file cells-13-01822-s001.zip › cells-3279020-supplementary.pdf]

Simultaneous stimulation of Peripheral Blood Mononuclear Cells with CpG ODN2006 and α-IgM antibody leads to strong immune responses in monocytes without affecting B cell activation

Leonie Fleige[1], Silvia Capellino[1*]

[1]Department of Immunology, Research Group of Neuroimmunology, IfADo-Leibniz Research Centre for Working Environment and Human Factors, Ardeystraße 67, 44139 Dortmund, Germany; fleige@ifado.de

* Corresponding author. E-mail address: capellino@ifado.de.

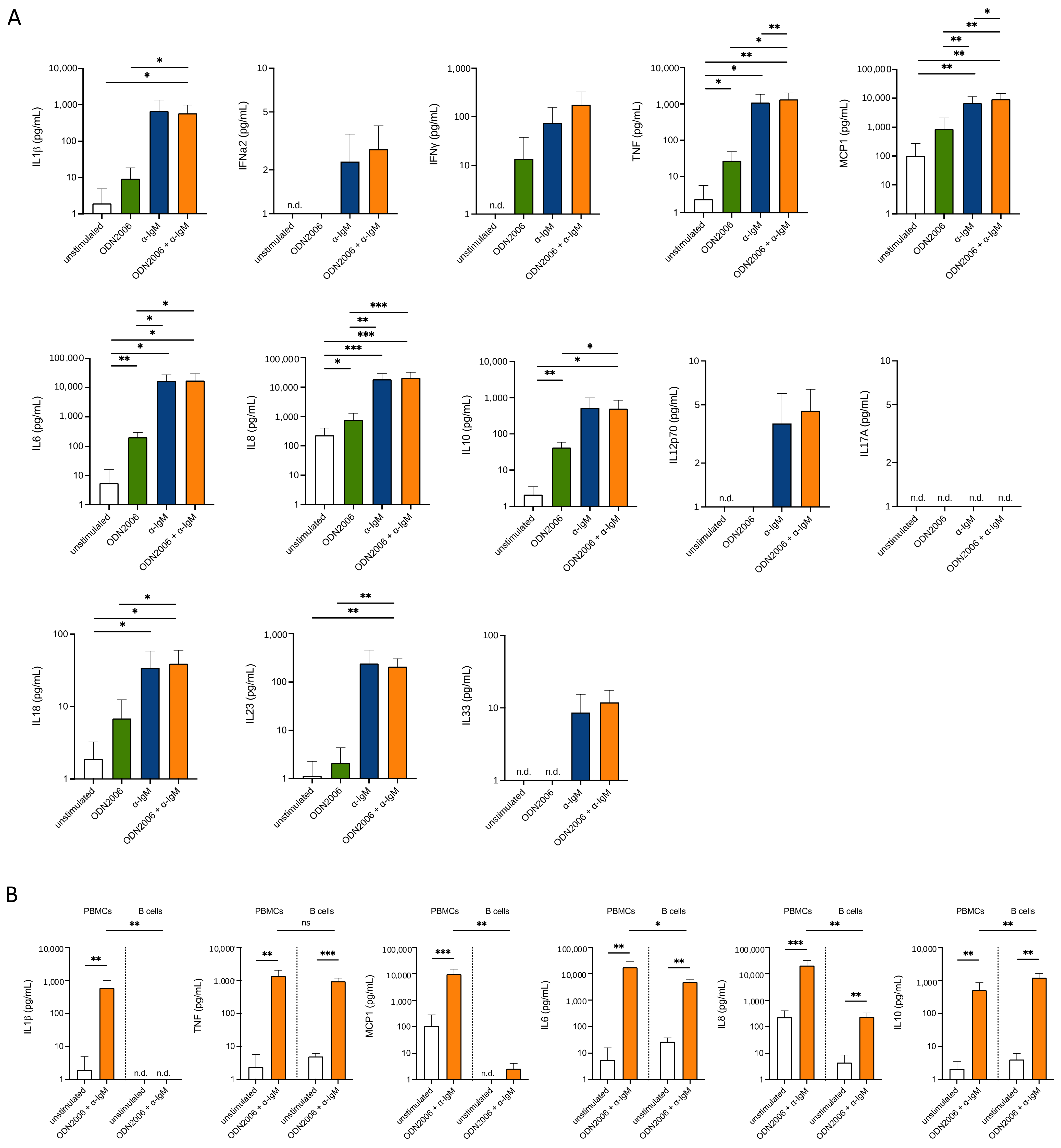

Supplementary Figure S1: **Stronger secretion of many cytokines after stimulation of mixed PBMCs with α-IgM compared to ODN2006. A**) Secreted IL1β, IFNα2, IFNγ, TNF, IL10, IL12p70, IL17A, IL18, IL23, IL33 (measured via Legendplex, n=6) and MCP1, IL6, IL8 (measured via ELISA, n=9-11) in supernatant of mixed PBMCs after ODN2006, α-IgM or ODN2006 + α-IgM stimulation for 24 h. Data already displayed in Figure 1 and B. **B**) Bar diagram of secreted IL1β, TNF and IL10 (measured via Legendplex, PBMCs n=7, B cells n=5) and MCP1, IL6, IL8 (measured via ELISA, PBMCs n=7, B cells n=5) in supernatant of mixed PBMCs vs isolated B cells after 24 h of stimulation with ODN2006 and α-IgM. Data already displayed in Figure 1B and D. N.d.: not detectable. No statistical test was performed for cytokines whose level was not detectable in unstimulated sample. One-Way ANOVA with Geisser-Greenhouse correction and Tukey's multiple comparisons test was used for analysing data including more than two stimulation conditions (A). Paired t test was used for the comparison of unstimulated vs. stimulated conditions within the same cell type (B). For comparing data from PBMCs with data from B cells, unpaired t test was applied, since different donors were used (B); *p ≤ 0.05, **p ≤ 0.01, ***p ≤ 0.001.

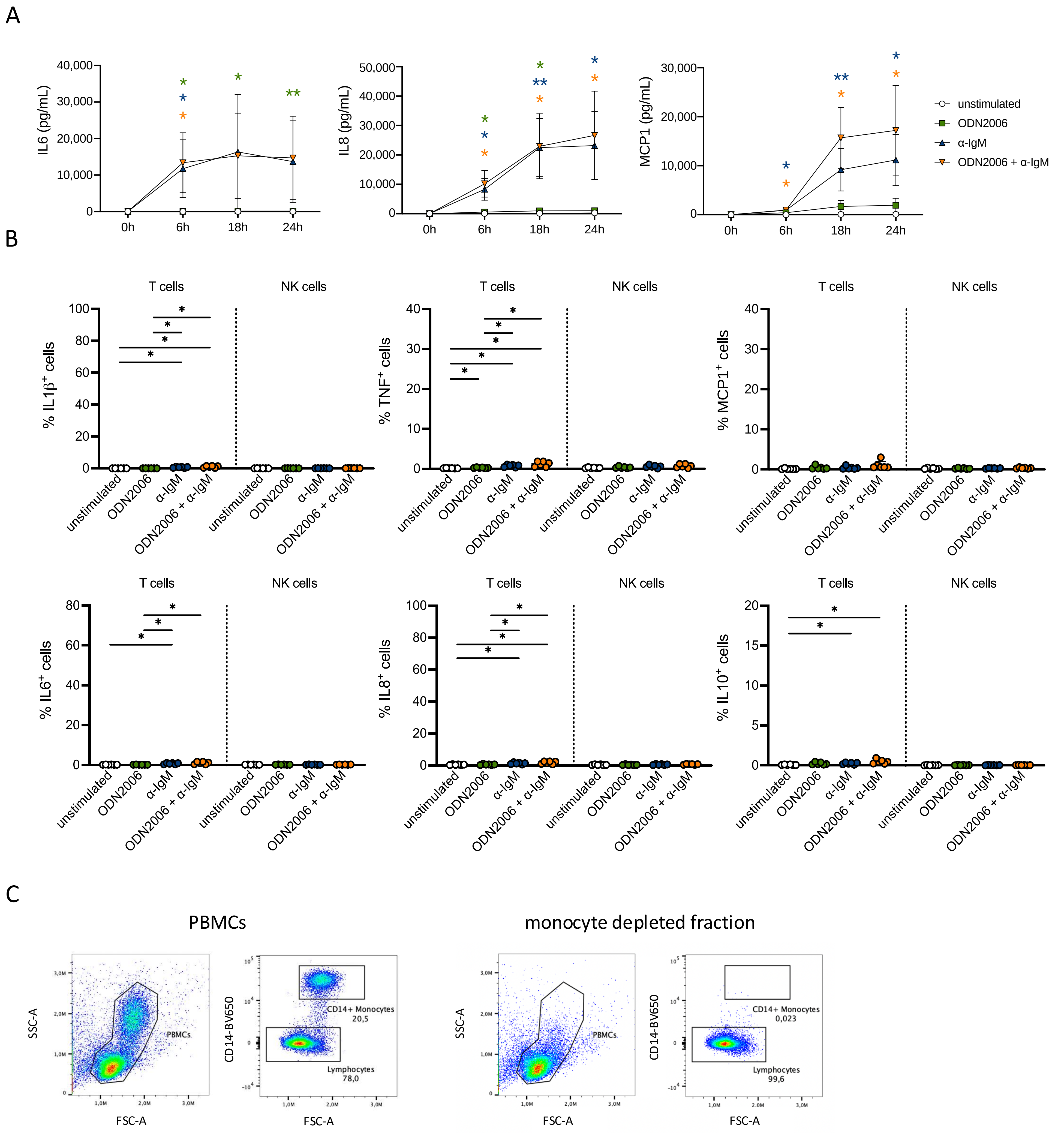

Supplementary Figure S2: Effects of ODN2006 and α-IgM on responses of monocytes mainly B cell independent. A) Time course measurement of secreted IL6, IL8, MCP1 in supernatant of mixed PBMC culture over 0, 6, 18 and 24 h via ELISA (n=6; statistics always refer to the unstimulated sample of the same timepoint). **B**) Intracellular cytokine staining for IL1β, TNF, MCP1, IL6, IL8 and IL10 in T cells and NK cells after ODN2006, α-IgM or ODN2006 + α-IgM stimulation for 6 h (IL1β, TNF, IL6, IL8) or 24 h (MCP1, IL10) via flow cytometry staining (n=6). **C**) Representative flow cytometry plots of mixed PBMCs and monocyte depleted fraction. Two-Way ANOVA with Geisser-Greenhouse correction and Tukey's multiple comparisons test was used for analysing data including more than two stimulation conditions and different time points (A). One-Way ANOVA with Geisser-Greenhouse correction and Tukey's multiple comparisons test was used for analysing data including more than two stimulation conditions but only one time point (B); *p ≤ 0.05, **p ≤ 0.01.

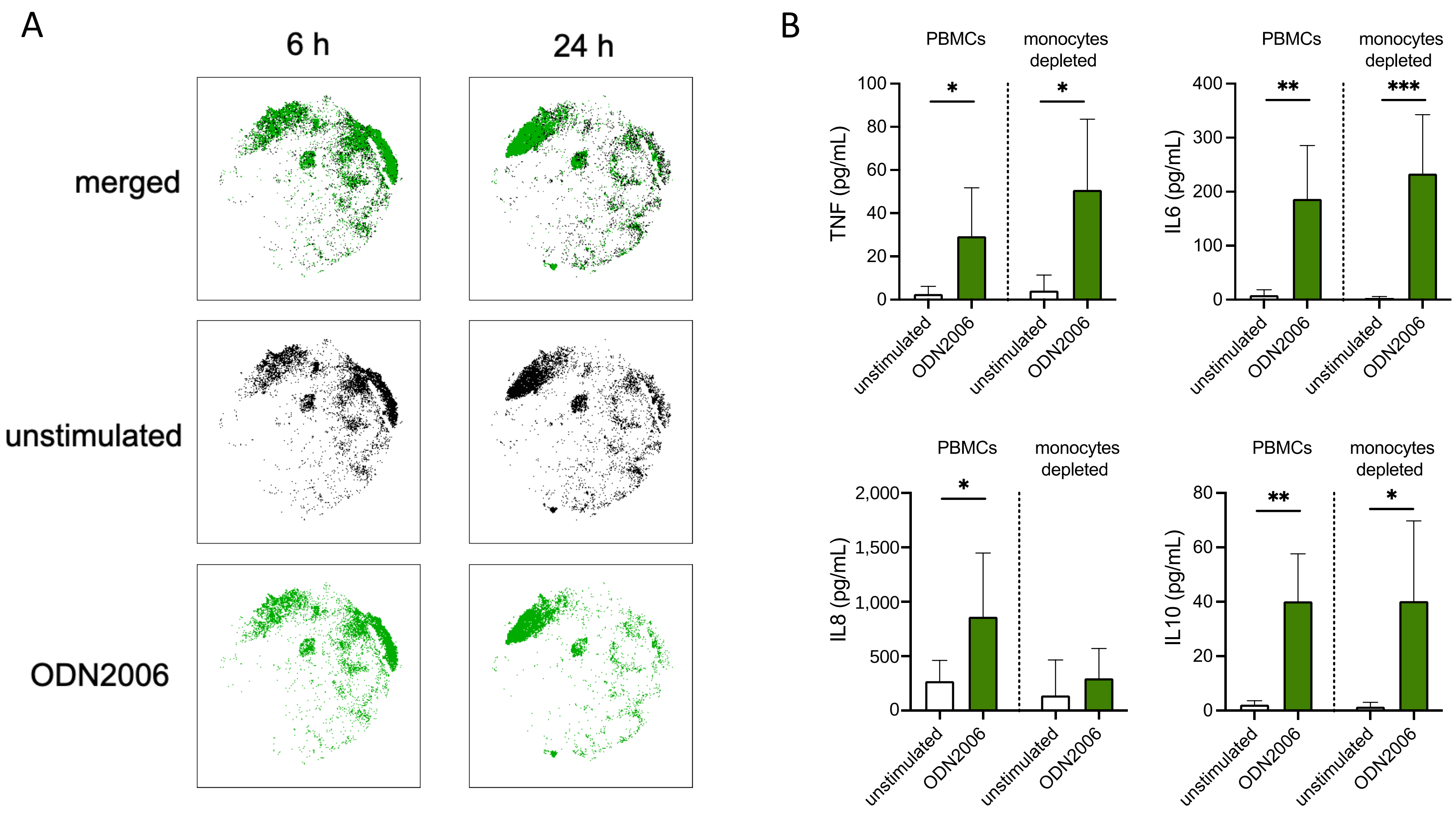

Supplementary Figure S3: ODN2006 did not influence the cytokine secretion of monocytes. A) t-SNE plots from samples stained for intracellular cytokines which were unstimulated (black) or stimulated with ODN2006 (green) for 6 h (left) and 24 h (right) after gating on CD14⁺ monocytes (n=6). **B**) Secreted TNF, IL6, IL8 and IL10 in supernatant of mixed PBMCs and monocyte depleted fraction after ODN2006 stimulation for 24 h measured with Legendplex for TNF and IL10 (n=6; statistical asterisks above bars refer to the unstimulated sample of the same cells) and ELISA for IL6 and IL8 (n=7-8; statistical asterisks above bars refer to the unstimulated sample of the same cells). Paired t test was used for the comparison of unstimulated vs. stimulated conditions within the same cell type as well as for comparing data from PBMCs with data from B cells (B); *p ≤ 0.05, **p ≤ 0.01, ***p ≤ 0.001.

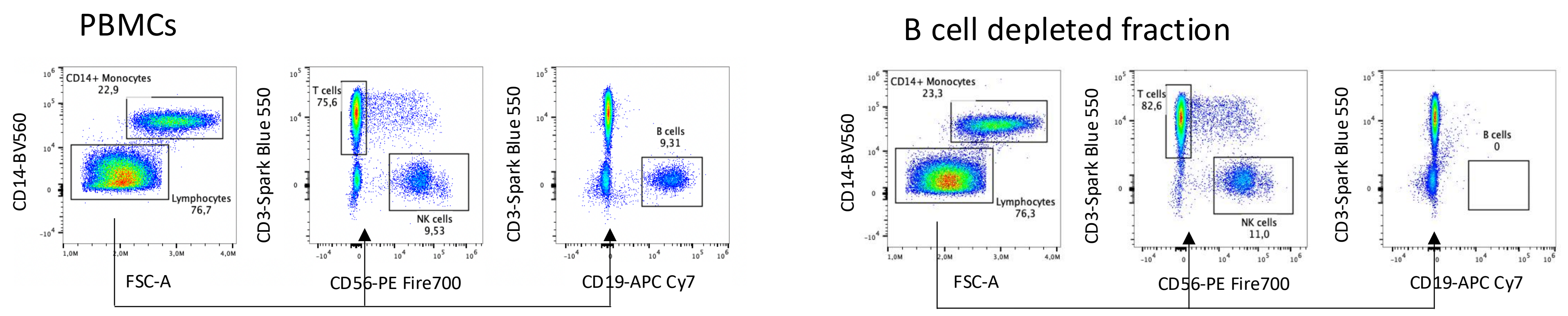

Supplementary Figure S4: Representative flow cytometry plots of mixed PBMCs (left) and B cell depleted fraction (right).

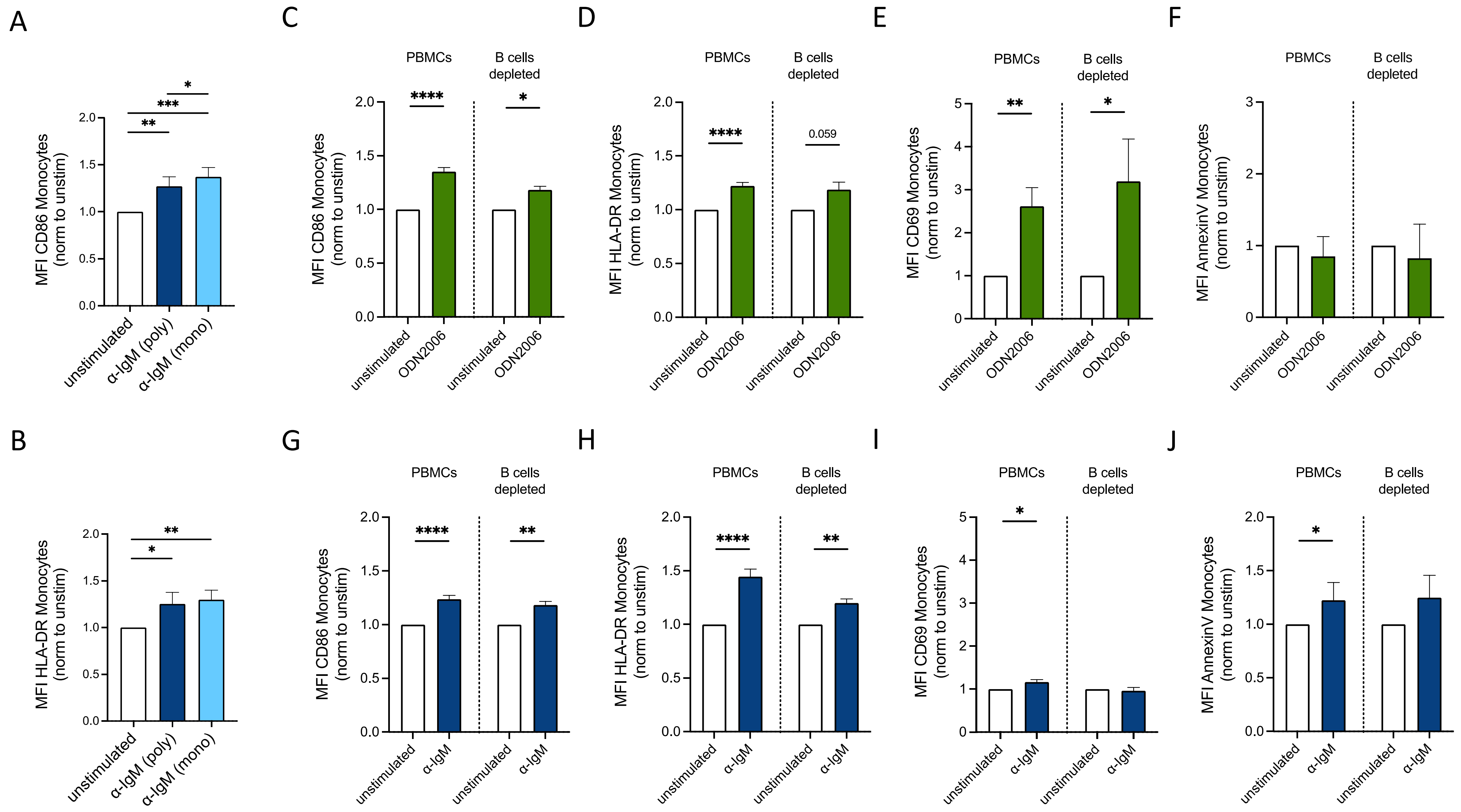

Supplementary Figure S5: ODN2006 and α-IgM affected activation marker expression on monocytes independently of B cells. **A-B**) Expression of CD86 (A) and HLA-DR (B) on monocytes after 6 h of stimulation with monoclonal and polyclonal α-IgM in culture of mixed PBMCs (n=6). **C-F**) Expression of CD86 (C), HLA-DR (D) and CD69 (E) on monocytes after 6 h and AnnexinV on monocytes after 24 h (F) of ODN2006 stimulation in culture of mixed PBMCs vs B cell depleted fraction (n=6-12). **G-J**) Expression of CD86 (G), HLA-DR (H) and CD69 (I) on monocytes after 6 h and AnnexinV on monocytes after 24 h (J) of α-IgM stimulation in culture of mixed PBMCs vs B cell depleted fraction (n=6-12). One-Way ANOVA with Geisser-Greenhouse correction and Tukey's multiple comparisons test was used for analysing data including more than two stimulation conditions (A-B). Paired t test was used to compare two conditions (C-J); $*p \leq 0.05$, $**p \leq 0.01$, $***p \leq 0.001$, $****p \leq 0.0001$.
